# *Selaginelladianzhongensis* (Selaginellaceae), a new spikemoss from China

**DOI:** 10.3897/phytokeys.118.30375

**Published:** 2019-03-06

**Authors:** Aleksandr Petrovich Shalimov, Yan-Mei Zhu, Meng-Hua Zhang, Xian-Chun Zhang

**Affiliations:** 1 State Key Laboratory of Systematic and Evolutionary Botany, Institute of Botany, Chinese Academy of Sciences (CAS), Beijing 100093, China State Key Laboratory of Systematic and Evolutionary Botany, Institute of Botany, Chinese Academy of Sciences Beijing China; 2 University of Chinese Academy of Science, Beijing, 100049, China University of Chinese Academy of Science Beijing China

**Keywords:** Lycophytes, *
Selaginella amblyphylla
*, taxonomy, Yunnan, *rbcL, atpI, psbA*

## Abstract

A new species of spikemoss from Yunnan Province of China, *Selaginelladianzhongensis*, is described and illustrated based on evidence from gross morphology, micromorphology and molecular phylogeny. *S.dianzhongensis* is most similar to *S.amblyphylla* in its habit of creeping stem, leaf size, and obviously dimorphic sporophylls, but is distinct by its ventral leaves ovate-oblong, subcordate at base, basiscopic base entire, axillary leaves ovate and decurrent at base. Molecular phylogeny analysis of three chloroplast gene regions (*rbcL, atpI, psbA*) shows that *S.dianzhongensis* forms an independent branch with strong support which is distantly related to *S.amblyphlla* and *S.kurzii*, but sister to *S.bodinieri* which is quite different in habitat of erect or ascending stem and rhizophores restricted to the lower part, and slightly dimorphic sporophyllus.

## Introduction

The initial critical taxonomic revision of Chinese *Selaginella* was published by Alston (1934), who recognized 41 species from China. In the Flora Reipublicae Popularis Sinicae and Flora of China (Zhang 2004, Zhang et al. 2013) the numbers of recognized species increased to 64 and 72, respectively. Recently, several new species and new records have been reported from China (Zhou et al. 2015a, Zhang and Sun 2015, Wu et al. 2017). Yunnan province is one of the species diversity centers of plants in China, with more than 53 species of *Selaginella* (Chu, 2006).

During field trips in Yunnan, we collected an unknown *Selaginella* species from Yimen County in central Yunnan. Morphology characters show it is similar to *S.amblyphylla* Alston, but phylogenetic analysis based on three chloroplast gene regions show it is close to *S.bodinieri* Hieron. Both molecular and morphology data support the taxon as a new species, which is described and illustrated here.

The new species belongs to subgenus Heterostachys in the classification system of Jermy (1986), and sect. Heterostachys of subgenus Heterostachys in the molecular-phylogeny classification proposed by Zhou and Zhang (2015), but this was rejected in the more robust molecular phylogenetic analysis based on chloroplast and nuclear genes by Weststrand and Korall (2016) that only recognized a broad subgenus Stachygynandrum.

## Material and methods

Herbarium specimens, silica gel and living materials were collected from Longquan Forest Park of Yimen (Yunnan province), in evergreen forest at 24°40'86"N, 102°08'27.86"E. Herbarium specimens are preserved in PE, and compared with similar species. The morpho-photographs of the plants were taken with a Nikon DXM 1200F camera connected to a stereomicroscope (Nikon SMZ 1000) and computer, measurements were done by D 3.10 (http:// www.nikoninstruments.com). The application ImageJ (https://imagej.nih.gov/ij/) was used to measure morphological characteristics (such as axillary, dorsal, ventral leaves, stems and strobili).

For study of spore morphology, scanning electron microscopy (SEM) was used. The spores were taken from mature sporangia and fixed on double line tape, and then covered with gold-palladium mixture. Spores were photographed and measured under different magnifications using a Hitachi S-4800 at 10–20 kV.

In this study, we sampled 28 taxa, representing almost all Chinese species with dimorphic strobili. Total genomic DNA was isolated from silica-dried material using the Plant Genomic DNA Kit (Tiangen Biotech, Beijing, China) following the manufacturer’s protocols. For each species, we attempted to amplify three chloroplast gene regions (*rbcL, atpI, psbA*) for the possible new taxa and its putative closely related taxa. These three regions were amplified with newly designed primers *rbcL* 192F (5’CACGTGGACTACCGTTTGGA3’) and 1324R (TACCCTCAAGAGCGGGATCA3’), *atpI* 119F (5’CYCAGGTTCATGGACAAGTAC3’) and *atpI* 540R (5’GRGTATYGGGGTTGGTTG3’), *psbA* 169F (5’CACGTGGACTACCGTTTGGA3’) and *psbA* 1026R (5’ATCTRGWGGGAAGTTGTGAGC3’), respectively. According to recent classification of *Selaginella* (Zhou and Zhang 2015; Weststrand and Korall 2016), we download 10 *rbcL* sequences of S.sect.Homostachys (*S.laxistrobila*, *S.helvetica*, *S.nipponica*), S.sect.Auriculate (*S.remotifolia*, *S.kraussiana*), S.sect.Oligomacrosporangiatae (*S.braunii*, *S.uncinata*, *S.delicatula*), and S.subg.Selaginoides (*S.deflexa*, *S.selaginoides*) from Genbank as outgroups. The 25 μL volume polymerase chain reactions (PCRs) contained 1 μL of plant DNA, 2.5 μL dNTPs, 1 μL each primer, 0.15 μL Taq polymerase (Takara Biotechnology Co., Dalian, China), 16.85 μL ddH_2_O. The PCR amplification profiles were identical for the three fragments: one cycle at 94 °C for 2 min; 35 cycles at 94 °C for 30 s, 52 °C for 1 min, 72 °C for 1.5 min; and one cycle at 72 °C for 10 min. All PCR products were directly sequenced using ABI 3730XL analyzer (Applied Biosystems, Foster City, California, USA). Newly obtained sequences were assembled with ContigExpress and then aligned with the downloaded sequences using Clustal X v.1.83 (Thompson et al. 1997) followed by manual adjustment in BioEdit v.7.1.11(Hall 1999). We have deposited all sequences into GenBank.

Phylogenetic tree of combined dataset (*rbcL+atpI+psbA*) was constructed using maximum likelihood (ML) and Bayesian inferences (BI). jModelTest 0.1.1 (Posada 2008) was used to select the appropriate substitution model for ML and BI analyses. The ML analysis was performed on the XSEDE online computing cluster accessed via the CIPRES Science Gateway (http://www.phylo.org) using RAxML-HPC2 v.8.2.8 (Stamatakis 2014), with 1000 bootstrap replicates under the GTRGAT model. Bayesian analyses and posterior probability (PP_BI_) calculation were conducted in Mr-Bayes 3.2.6 (Ronquist et al. 2012) implemented on the CIPRES Science Gateway Portal (Miller et al. 2010). We ran four chains of the Markov chain Monte Carlo (MCMC), sampling one tree every 100 generations for 1,000,000, starting with a random tree. Bayesian posterior probabilities (PP) were calculated as the majority consensus of all sampled trees with the first 25% discarded as burn-in.

## Results

### Taxonomic treatment

#### Selaginella dianzhongensis

Taxon classificationPlantaeSelaginellalesSelaginellaceae

X.C.Zhang sp. nov.

urn:lsid:ipni.org:names:60478269-2

Figures 1 , 2

##### Diagnosis.

The new species resembles *Selaginellaamblyphylla* in habit and gross morphology, but it is different in stems and branches reddish (vs. stramineous in *S.amblyphylla*), ventral leaves ovate-oblong, 1.1–2.2 × 0.4–0.8 mm (vs. oblong, 2–3 × 0.6–1.2 mm), base subcordate, basiscopic margin not ciliolate (vs. rounded and margin sparsely ciliolate); dorsal leaves oblique subcordate or cordate at base (vs. obliquely cordate), margin with rather long cilia (vs. denticulate or ciliolate); axillary leaves ovate and decurrent at base (vs. ovate or triangular and obtuse to decurrent at base); strobili 3.2–4.0 × 2.3–3.5 mm (vs. 3.5–10 × 3.2–4.4 mm), ventral sporophylls margin ciliolate, dorsal sporophylls margin denticulate (vs. both sporophylls margin ciliolate).

**Figure 1.**
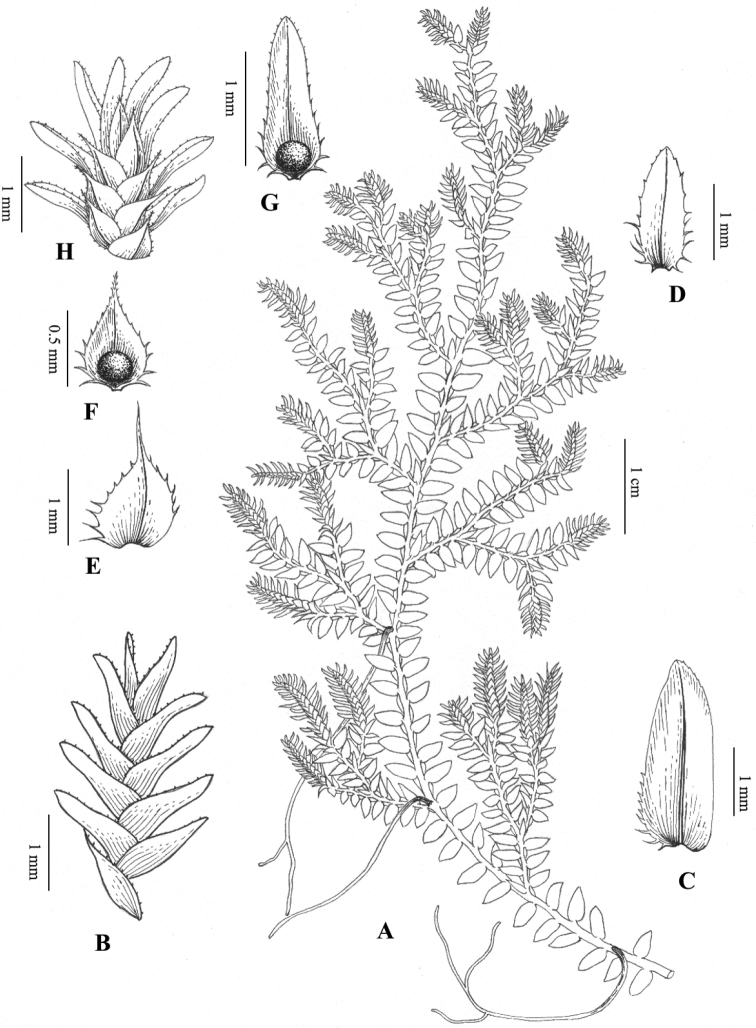
*Selaginelladianzhongensis* X.C.Zhang, sp. nov. **A** habit **B** adaxial view of strobilus **C** ventral leaf **D** axillary leaf **E** dorsal leaf **F** adaxial view of lower sporophyll **G** adaxial view of upper sporophyll **H** abaxial view of strobilus (Illustration made by Huixia Dong).

**Figure 2.**
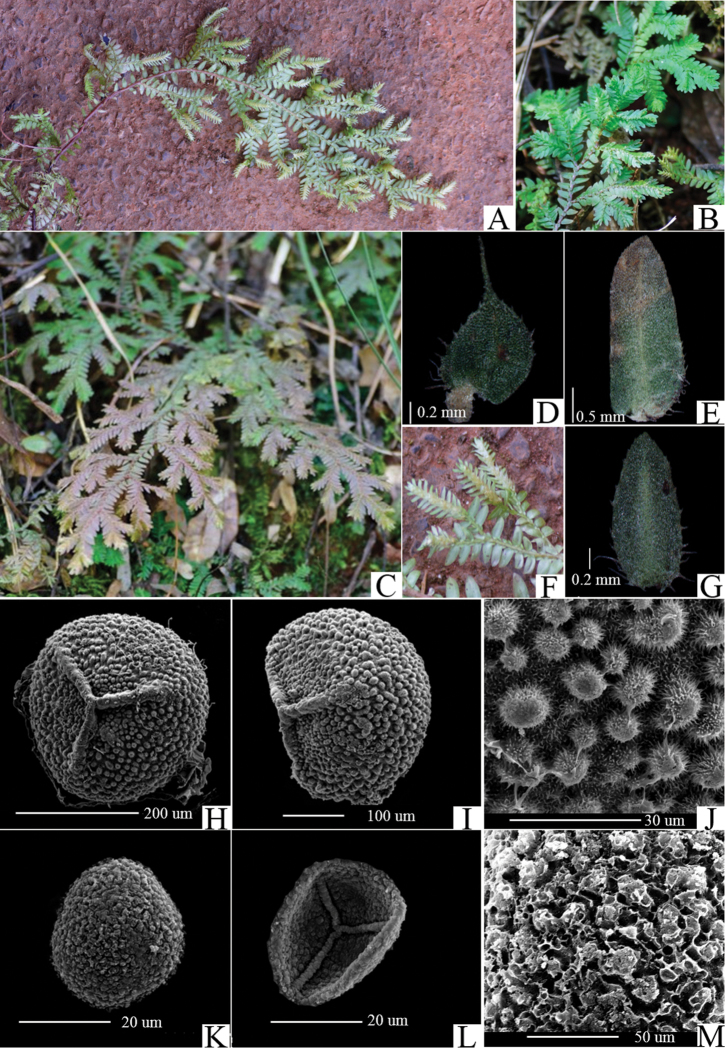
*Selaginelladianzhongensis* X.C.Zhang, sp. nov. **A** individual **B** portion of plant **C** habit **D** dorsal leaf **E** ventral leaf **F** strobili **G** axillary leaf **H** proximal surface of megaspore **I** distal surface of megaspores **J** portion of megaspore surface enlarged to show infrastructural detail **K** distal surface of microspore **L** proximal surface of microspore **M** portion of microspore surface enlarged to show infrastructural detail surface (Taken from *Yan-Mei Zhu 8158* (PE)).

##### Type.

CHINA, Yunnan Province, Yimen County, Longquan Forest Park, 9 Feb 2017, *Yan-Mei Zhu 8158* (Holotype, PE!; isotype PE).

##### Description.

Plants terrestrial, evergreen. Main stems reddish, creeping or suberect, stems 15–25 cm long, 0.6–1.0 mm in diam., branches stramineous. *Rhizophores* restricted in basal part of main stems or at intervals throughout length of creeping stem and branches, borne on ventral side in axils of branches. *Main stems* branched from near base, slightly sulcate, primary branches 0.7–1.5 cm apart, secondary branches once or twice pinnately branched, leafy portion of main stem including leaves 6–8 mm wide at middle, ultimate branches 4–7 mm wide including leaves. *Axillary leaves* on main stems and branches symmetrical, ovate, 1.1–2.2 × 0.4–0.8 mm, at base decurrent, margin sparsely long ciliate at base, ciliolate or denticulate to the apex, apex acute. *Dorsal leaves* ± symmetrical, on main stems distantly, on branch imbricate, on main stem larger than on branches, ovate to broadly ovate, 1.2–1.9 × 0.5–1.0 mm, slightly carinate on abaxial surface, in base oblique subcordate or cordate, not peltate, margin ciliolate at base, apex long aristate, arista 0.3–0.6 mm long. *Ventral leaves* asymmetrical, slightly distant, on main stem bit larger than those on branches, ovate-oblong, 1.7–3.9 × 0.7–1.6 mm, in base subcordate; *acroscopic base* slightly overlapping stems and branch, margin long ciliolateat base, denticulate to the apex, *basiscopic bases* free from stems, margin entire, apex acute. *Strobili* compact, solitary, terminal on branch tips, dorsiventrally complanate, 3.2–4.0 × 2.3–3.5 mm. *Sporophylls* strongly dimorphic: dorsal sporophylls ovate-lanceolate, margin denticulate, apex acuminate, with sporophyll-pteryx slightly incomplete, margin denticulate, ventral sporophylls ovate-lanceolate, ascending, carinate, margin ciliolate, apex cuspidate. Megaspores white-yellow; proximal and distal surfaces verrucate, micro-sculpture densely echinate. Microspores yellowish, proximal and distal surfaces irregularly verrucate, micro-sculpture with interconnected and blunt spinules.

##### Etymology.

Dianzhong means central Yunnan in Chinese: the type locality (Yimen) is in the central Yunnan area which is centered on the Provincial capital city Kunming.

##### Distribution and habitat.

**Figure 3.**
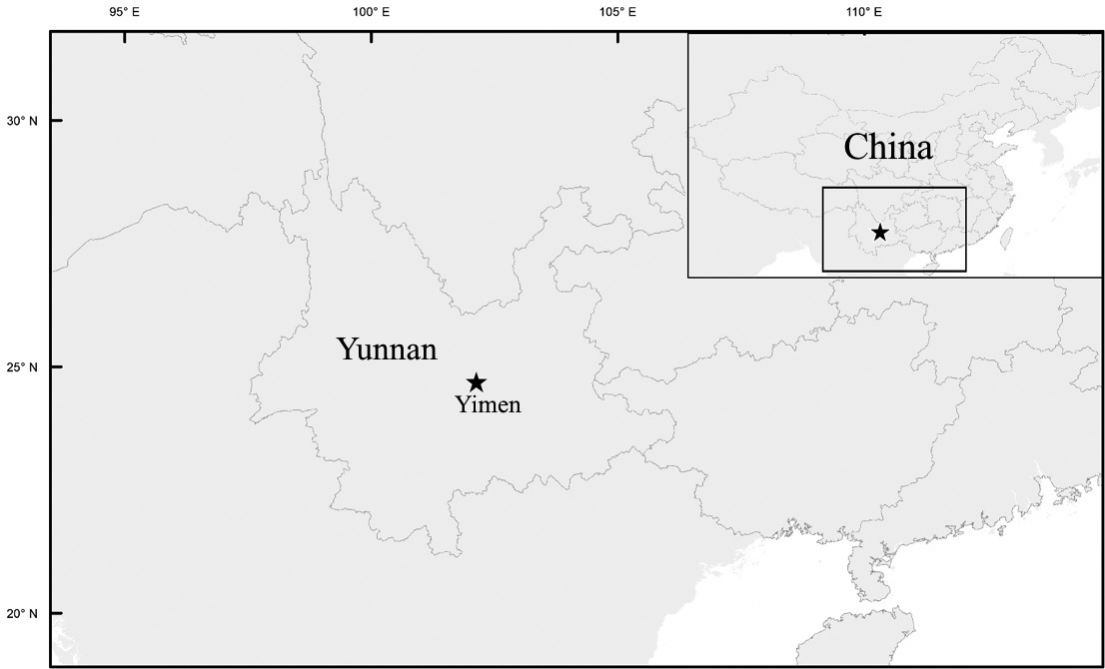
Distribution of *Selaginelladianzhongensis* X.C.Zhang, sp. nov.

*Selaginelladianzhongensis* is known only from Yimen county, Yunnan, growing on mossy soils in a mixed evergreen forest, at ca. 1576 m a.s.l. (Fig. 3).

##### Conservation status (VU).

*Selaginelladianzhongensis* is known only from one locality inside the Longquan Forest Park in Yimen county, with more than 300 individuals. This park has a heavy recreational load and human pressure, and there are no specific measures to protect the habitats. Considering the restricted distribution and plausible threats, we tentatively assessed *Selaginelladianzhongensis* as vulnerable (VU) according to the IUCN (2018) categories and criteria.

##### Specimens examined.

***Selaginellaamblyphylla*** Alston: **THAILAND**: *Payar*, Doi Angka, *H. M. Smith 357* – BM [BM000779901, image online!] (holotype), – US [US00134348, image online!], MICH [MICH1191432, image online!], GH [GH00022032, image online!] (isotypes), Doi Cheng Dao, 4800 ft., on rock, Oct–Nov 1922, *E. Smith 1262* – SING [!, image] (paratype); Udawn, 900–1400 m, *Tagawa c.s. T-1816* [PE01622140]; **CHINA**:Yunnan, Mengla County, *B. G. Li 48926* [PE00405395]; Zhenkang *W. M. Chu et al. 15204* [PE00405401]; Yunnan, Gengma, *W. M. Chu et al. 15279* [PE00405408]; Guangxi, Lingui, *J. X. Zhong 808194* [PE01593730]; Yunnan, Simao (Szemao), ravine, 4000 ft., *A. Henry 13529* – NY [NY00127369, image online!] (paratype). ***Selaginellabodinieri*** Hieron. Guizhou, Bijie, *X. C. Zhang et al*. 6842 [PE 01962745]; Guangxi, Fengshan, Alt. 750 m, *X. C. Zhang 1272* [PE 00405447]; ***Selaginellakurzii*** Baker, Yunnan, Cangyuan, *J. C. Zhao 2000-13* [PE 00405796]; Luquan, *W. M. Chu 1649* [PE 00405795]; Mengla (Cha-li-Hsien), alt. 950 m, *C. W. Wang 77750* [PE 01634093].

### Phylogenetic Analysis

**Figure 4.**
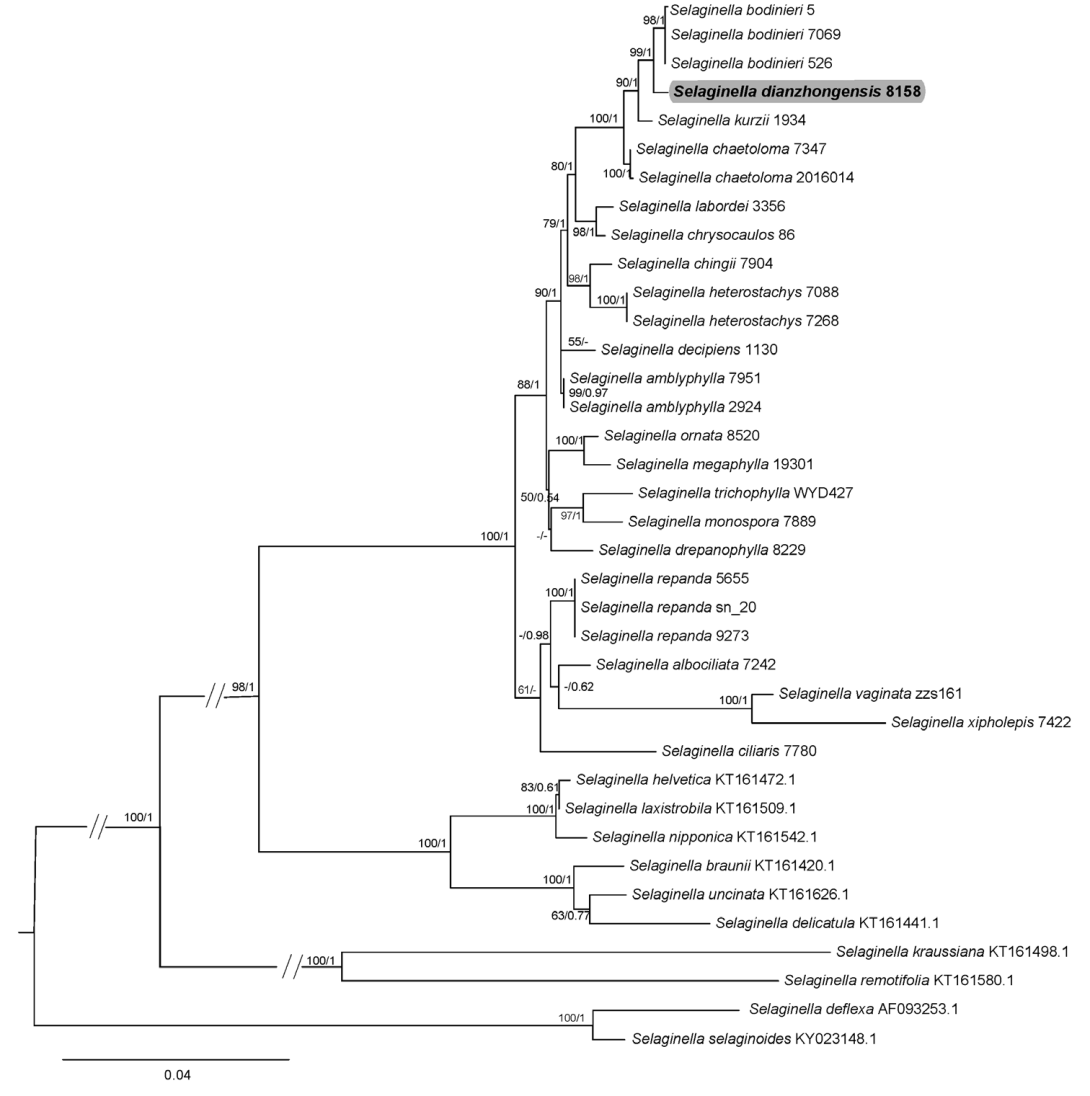
The 50% majority rule consensus tree derived from maximum likelihood showing the position of *Selaginelladianzhongensis*. Support values (BSML/PP_BI_) are shown above the main braches; the dash (–) indicates BS < 50%. The new species is shown in bold.

The combined data matrix included up to 2045 nucleotides for each of the 37 taxa with 374 parsimony informative sites (374/2045 = 18.29%), consistency index (CI) = 0.66, retention index (RI) = 0.80, when the gaps were treated as missing data. The tree recovered from maximum likelihood (ML) and Bayesian inferences (BI), with bootstrap values (BS) of ML and Bayesian posterior probabilities (PP) for each clade is shown in Fig. 4. The new species sampled from Yimen clustered with *Selaginellabodinieri* with strong support (BS_ML_ = 99; PP_BI_ = 1.0), but the new species is quite similar to the *S.amblyphylla* rather than *S.bodinieri* in morphological characters.

## Discussion

Morphologically, the shape and margin of ventral and dorsal leaves of *Selaginelladianzhongensis* is most similar to *S.amblyphylla*. But the axillary leaves of the former are ovate, 1.1–2.2 × 0.4–0.8 mm, margin with a few long cilia (vs. ovate or triangular, 2–3 × 0.6–1.2 mm, and denticulate at margin in *S.amblyphylla*). Ventral leaves of the former are ovate-oblong, apex acute (vs. oblong and obtuse or subacute at apex in *S.amblyphylla*), basiscopic margin entire and not ciliolate (vs. basiscopic margin sparsely ciliolate at base in *S.amblyphylla*), acroscopic base of ventral leaf long ciliolate (vs. shortly ciliolate in *S.amblyphylla*).

Molecular data showed that *S.dianzhongensis* clustered with two other species: *S.kurzii* Baker and *S.bodinieri*.

*S.dianzhongensis* is indeed similar to *S.kurzii*, but fertile branches are not erect (vs. erect in *S.kurzii*), dorsal leaves are ovate to broadly ovate with arista at apex (vs. ovate or ovate-elliptic, acuminate or aristate at apex), and ventral leaves are ovate-oblong, basiscopic margin entire and not ciliolate (vs. ovate-triangular, basiscopic margin entire or with 1 or 2 cilia at base).

*Selaginellabodinieri* is widely distributed in the limestone areas from central to southwestern China: main differences between this species (and the other ones mentioned above) and *S.dianzhongensis* are reported in the key below, and in Table 1.

Finally, mega- and microspores of *S.dianzhongensis* are morphologically different from the spores of similar species studied by Zhou et al. (2015b). Megaspores of *S.dianzhongensis* have verrucae on proximal and distal side; micro-sculptures of megaspores are densely echinate on both sides (Fig. 2 H–J). Microspores of *S.dianzhongensis* are verrucate, with blunt spinules (Fig. 2 K–M). Morphological comparison of mega- and microspores between *S.dianzhongensis* and closely related species is presented in Table 2.

**Table 1.** Morphological characters of *Selaginellaamblyphylla*, *S.bodinieri*, *S.dianzhongensis* and *S.kurzii*.

Characters	* S. amblyphylla *	* S. bodinieri *	* S. dianzhongensis *	* S. kurzii *
Main stems	creeping, up to 35 cm	erect or ascending, (15–)30–40(–50) cm	creeping, 15–25 cm	erect or ascending, 10–20 cm
Axillary leaves	ovate or triangular, 2–3 × 0.6–1.2 mm	ovate or triangular, 2–3.2 × 0.9–1.6 mm	ovate, 1.1–2.2 × 0.4–0.8 mm	ovate or ovate-lanceolate, 1–2.5 × 0.6–1.6 mm
Base of axillary leaf	denticulate	denticulate or ciliolate	with a few long cilia	long ciliolate
Dorsal leaves	ovate-lanceolate or ovate, 1.4–2.2 × 0.4–0.8 mm	obliquely ovate, 2.4–3.4 × 1.2–1.8 mm	ovate to broadly ovate, 1.2–1.9 × 0.5–1.0 mm	ovate or ovate-elliptic, 1–1.2 × 0.4–0.8 mm
Base of dorsal leaf	obliquely cordate, denticulate to ciliolate	obliquely cordate, denticulate or ciliolate	obliquely subcordate or cordate, ciliolate	subcordate or obtuse, ciliolate,
Apex of dorsal leaf	aristate, arista ca. 1 mm long	acuminate, aristate, or cuspidate	aristate, arista 0.3–0.6 mm long	acuminate or aristate, arista 0.3–0.6 mm long
Ventral leaves	oblong, 2.2–3.5 × 1.6–2 mm, apex obtuse or subacute	oblong-ovate or oblong, 3.4–4.4 × 1.6–2.2 mm, acute or obtuse	ovate-oblong, 1.7–3.9 × 0.7–1.6, apex acute	ovate-triangular, 1.6–3.8 × 0.6–1.6 mm, apex acute or acuminate
Acroscopic base of ventral leaf	shortly ciliolate in basal portion, elsewhere entire	denticulate or ciliolate	long ciliolate	rather long ciliolate at base, subentire upward
Basiscopic base of ventral leaf	sparsely ciliolate at base	entire	slightly auriculate in base, margin entire	entire or with 1 or 2 cilia at base
Strobili	3.5–10 × 3.2–4.4 mm	4–16 × 1.4–2.4 mm	3.2–4.0 × 2.3–3.5 mm	6–8 × 2–3 mm

**Table 2.** Morphological characters of mega- and microspores of *Selaginellaamblyphylla*, *S.bodinieri*, *S.dianzhongensis* and *S.kurzii*.

**Characters**	*** S. amblyphylla ***	*** S. bodinieri ***	*** S. dianzhongensis ***	*** S. kurzii ***
**Megaspores**				
Megaspores: proximal and distal surfaces	verrucae	verrucae	verrucae	verrucae
Megaspores: micro-sculptures	vermiculate	spinulose	densely echinate	vermiculate
**Microspores**				
Microspores: proximal and distal surfaces	irregularly sized and spaced verrucae	irregularly sized and spaced verrucae	irregular size verrucae	irregularly sized and spaced verrucae
Microspores: micro-sculptures	dense spinules	not present	blunt spinules	dense spinules

### Key to the *S.dianzhongensis* and related species in Yunnan

**Table:** 

1	Main stems creeping or suberect, fertile stems not erect	**2**
–	Main stems creeping, fertile stems erect	*** S. kurzii ***
2	Ventral leaves strongly overlapping stems and branches, basiscopic base exauriculate and margins ciliolate	*** S. amblyphylla ***
–	Ventral leaves not overlapping stems and branches, basiscopic base slightly auriculate and margins entire or ciliolate	**3**
3	Plants 40–50 cm long, main stems unbranched in lower to middle part, with stolons at bases, basiscopic base of ventral leaves slightly auriculate, acroscopic base denticulate or ciliolate at margins	*** S. bodinieri ***
–	Plants about 15–25 cm long, main stems branched from near base, rhizophores restricted to lower part of stem, basiscopic base ventral leaves entire, acroscopic base rather long ciliolate at margins	*** S. dianzhongensis ***

## Supplementary Material

XML Treatment for Selaginella dianzhongensis

## Appendix

Information of the plant materials used in this study is presented in the following order: taxon name, locality (if available), collection number (if available), *rbcL* GenBank accession number, *atpI* GenBank accession number, *psbA* GenBank accession number references (if available). –, sequences not available. *, sequences downloaded from NCBI.

***Selaginellaalbociliata*** P.S. Wang, Guizhou, China, Zhang X.-C. 7242.(PE), MH814882, MH814826, MH814854. ***Selaginellaamblyphylla*** Alston, Yunnan, China, Zhang X.-C. 2924.(PE), MH814883, MH814827, MH814855. ***Selaginellaamblyphylla*** Alston, Yunnan, China, Zhang X.-C. 7951.(PE), MH814884, MH814828, MH814856. ***Selaginellabodinieri*** Hieron., Chongqing, China, Zhang X.-C. 5.(PE), MH814885, MH814829, MH814857. ***Selaginellabodinieri*** Hieron., Sichuan, China, Zhang X.-C. 526.(PE), MH814886, MH814830, MH814858. ***Selaginellabodinieri*** Hieron., Guizhou, China, Zhang X.-C. 7069.(PE), MH814887, MH814831, MH814859. ***Selaginellachaetoloma*** Alston, Guizhou, China, Guo Z.-Y. 2016014.(PE), MH814888, MH814832, MH814860. ***Selaginellachaetoloma*** Alston, Guizhou, China, Zhang X.-C. 7347.(PE), MH814889, MH814833, MH814861. ***Selaginellachingii*** Alston, Guangxi, China, Zhang X.-C. 7904.(PE), MH814890, MH814834, MH814862. ***Selaginellachrysocaulos*** (Hook. & Grev.) Spring, Sichuan, China, Zhang X.-C. 86.(PE), MH814891, MH814835, MH814863. ***Selaginellaciliaris*** (Retz.) Spring, Yunnan, China, Zhang X.-C. 86.(PE), MH814892, MH814836, MH814864. ***Selaginelladecipiens*** Warb., Guangxi, China, Zhang X.-C. 7253.(PE), MH814893, MH814837, MH814865. ***Selaginelladianzhongensis*** X.C.Zhang, Yunnan, China, Zhu Y.-M. 8158.(PE), MH814909, MH814853, MH814881. ***Selaginelladrepanophylla*** Alston, Yunnan, China, Zhang X.-C. 8229.(PE), MH814894, MH814838, MH814866. ***Selaginellatrichophylla*** K. H. Shing, Guizhou, China, Wu Y.-D. 427.(PE), MH814895, MH814839, MH814867. ***Selaginellaheterostachys*** Baker, Guizhou, China, Zhang X.-C. 7088.(PE), MH814896, MH814840, MH814868. ***Selaginellaheterostachys*** Baker, Guizhou, China, Zhang X.-C. 7268.(PE), MH814897, MH814841, MH814869. ***Selaginellakurzii*** Baker, Yunnan, China, Zhang X.-C. 1934.(PE), MH814898, MH814842, MH814870. ***Selaginellalabordei*** Hieron. ex Christ, Hubei, China, Zhang X.-C. 3356.(PE), MH814899, MH814843, MH814871. ***Selaginellamegaphylla*** Baker, Tibet, China, Jin X.-H. 19301.(PE), MH814901, MH814845, MH814873. ***Selaginellamonospora*** Spring, Guangxi, China, Zhang X.-C. 7889.(PE), MH814902, MH814846, MH814874. ***Selaginellaornata*** (Hook. & Grev.) Spring, Yunnan, China, Zhang X.-C. 8520.(PE), MH814903, MH814847, MH814875. ***Selaginellarepanda*** (Desv. ex Poir.) Spring, Yunnan, China, Zhang X.-C. 5655.(PE), MH814904, MH814848, MH814876. ***Selaginellarepanda*** (Desv. ex Poir.) Spring, Yunnan, China, Zhang X.-C. 9273.(PE), MH814905, MH814849, MH814877. ***Selaginellarepanda*** (Desv. ex Poir.) Spring, Yunnan, China, Li B.-G. sn_20.(PE), MH814906, MH814850, MH814878. ***Selaginellavaginata*** Spring, Shaanxi, China, Zhang Z.-S. 161.(PE), MH814907, MH814851, MH814879. ***Selaginellaxipholepis*** Baker, Guizhou, China, Zhang X.-C. 7422.(PE), MH814908, MH814852, MH814880. ***Selaginellabraunii*** Baker, voucher Zhang 1332 (PYU, CDBI), KT161420.1 *, -, -. ***Selaginelladelicatula*** (Desv. ex Poir.) Alston, voucher Gao & al. HGX10734 (CDBI), KT161441.1*, -, -. ***Selaginellahelvetica*** (L.) Link, voucher Zhou 093 (CDBI), KT161472.1*, -, -. ***Selaginellakraussiana*** (Kunze) A. Braun, voucher Zhou 062 (CDBI), KT161498.1*, -, -. ***Selaginellalaxistrobila*** K.H. Shing, voucher Chu & al. 24449 (PYU), KT161509.1*, -, -. ***Selaginellanipponica*** Franch. & Sav., voucher Zhou & al. DJY07479 (CDBI), KT161542.1*, -, -. ***Selaginellaremotifolia*** Spring, voucher Zhou 005 (PYU, CDBI), KT161580.1*, -, -. ***Selaginellauncinata*** (Desv. ex Poir.) Spring, voucher Zhang & Zhou DJY04101 (CDBI), KT161626.1*, -, -. ***Selaginelladeflexa*** Brack., AF093253.1*, -, -. ***Selaginellaselaginoides*** (L.) P. Beauv. ex Schrank & Mart., voucher S. Weststrand 104 (UPS), KY023148.1*, -, -.
